# Expression Pattern of Genes of RLR-Mediated Antiviral Pathway in Different-Breed Chicken Response to Marek's Disease Virus Infection

**DOI:** 10.1155/2013/419256

**Published:** 2013-04-08

**Authors:** Ze-Qing Feng, Ting Lian, Yong Huang, Qing Zhu, Yi-Ping Liu

**Affiliations:** ^1^College of Animal Science and Technology, Sichuan Agriculture University, Ya'an, Sichuan 625014, China; ^2^College of Veterinary Medicine, Sichuan Agriculture University, Ya'an, Sichuan 625014, China

## Abstract

It has been known that the chicken's resistance to disease was affected by chicken's genetic background. And RLR-mediated antiviral pathway plays an important role in detection of viral RNA. However, little is known about the interaction of genetic background with RLR-mediated antiviral pathway in chicken against MDV infection. In this study, we adopted economic line-AA broilers and native Erlang mountainous chickens for being infected with MDV. Upon infection with MDV, the expression of *MDA-5* was upregulated in two-breed chickens at 4, 7, and 21 d.p.i. It is indicated that MDA-5 might be involved in detecting MDV in chicken. Interestingly, the expression of *IRF-3* and *IFN-**β*** genes was decreased in spleen and thymus of broilers at 21 d.p.i, but it was upregulated in immune tissues of Erlang mountainous chickens. And the genome load of MDV in spleen of broiler is significantly higher than that in Erlang mountainous chickens. Meanwhile, we observed that the death of broiler mainly also occurred in this phase. Collectively, these present results demonstrated that the expression patters of *IRF-3* and *IFN-**β*** genes in chicken against MDV infection might be affected by the genetic background which sequently influence the resistance of chicken response to MDV.

## 1. Introduction

Innate immune system serves as the first line for detecting and defending against invading pathogens [1]. It detects pathogen associated molecular patterns (PAMP) by employing Pattern-Recognition Receptors (PRRs) and triggers the production of type I interferon for preventing viral replication and diffusion [2, 3]. PRRs are composed of toll-like receptors (TLRs), retinoic-acid-inducible-gene-I- (RIG-I-) like receptors (RLRs), NOD-like receptors (NLRs), and C-type lectin receptors (CLRs). RLRs, located in cytoplasm, consists of retinoic acid-induced gene-I (RIG-I) [4], melanoma differentiation associated gene-5 (MDA-5) [5], laboratory of genetics and physiology-2 (LGP-2) [6]. RIG-I and MDA-5 recognize different length of viral double-stranded RNA (dsRNA) by their RNA helicase domain [6, 7]. Additionally, *RIG-I* is capable of recognizing single-stranded RNA (ssRNA) containing 5′-triphosphate by its C-terminal regulator domain which inhibits the activation of RIG-I in the steady state [8–11]. Once RIG-I and MDA-5 bind with ssRNA or dsRNA derived from virus, it can activate downstream transcription factors such as NF-*κ*B, IRF-3, and IRF-7, then these transcription factors translocate from cytoplasm into nucleus and efficiently induce expression of genes encoding type I interferon [12–14]. 

It has been established that RLR-mediated innate immune plays a crucial role in human and mouse response to viral infection. Previous study indicated that the absence of *RIG-I* in chicken results in more susceptibility of chickens to influenza viruses than ducks [15]. Recently, *MDA-5* and *LGP-2* have been identified in chicken, and MDA-5 has been shown to be involved in sensing dsRNA and influenza A virus in chicken cell [16, 17]. However, the exact role of MDA-5 *in vivo* of chicken against virus infection has not been clarified in detail, and little study has been devoted to investigate the role of RLR-mediated antiviral pathways in chicken response to DNA virus infection.

Marek's disease (MD), which is caused by Marek's disease virus (MDV), is lymphoproliferative tumour disease in chickens, which clinically shows the immune suppression, polyneuritis, and formation of T-cell lymphoma in the visceral [18]. MDV belongs to *α*-herpesvirus subfamily owing to its molecular structure and genomic organization close to herpes simplex virus (HSV) [19–21]. Previous studies showed that expression of many proinflammatory cytokine genes, including *IFN-*α*, IFN-*γ*, iNOS, IL-1*β*, IL-6* and *IL-18*, have been enhanced in chicken following infection with MDV [22–25]. Additionally, the changes of these cytokines expression *in vivo* were influenced by genetic background of chicken and virulence of MDV [26–28]. Meanwhile, the expression of *TLR-3* and *TLR-7* genes was induced in the lungs of chicken response to MDV infection [23]. These results impel us to determine whether RLR-mediated innate immune pathways participate in chickens immune against MDV. Meanwhile we also want to know whether the expression of gene of RLR-mediated innate immune pathway is affected by genetic background.

 To address these objectives, two-breed chickens including economic line-AA broilers and native Erlang mountainous chickens were chose for infection with MDV. Then the expression of *MDA-5, IRF-3, IFN-*α** and **β** gene in the immune organ at 4, 7, and 21 d.p.i were measured by real-time PCR. These results will make us to understand the roles of genetic background and RLR-mediated immune pathway in chicken response to MDV infection.

## 2. Materials and Methods

### 2.1. Experimental Animals and Virus

Fertilized eggs of Erlang mountainous chickens and AA broilers were obtained from Long-Sheng Company and Zheng-Da Company of China, respectively. All eggs were hatched at incubation room of Long-Sheng Company; chickens hatched were unvaccinated and housed in the isolation laboratory of veterinary hospital of Sichuan agricultural University. All chickens used in the study were approved by the Sichuan Agricultural University Animal Care and Use Committee.

The virulent MDV J-1 strain used in the study was purchased from institute of animal and veterinary in Beijing. The virus was always kept in the liquid nitrogen until used.

### 2.2. Experimental Design and Samples Collection

One hundred and 3 days posthatched Erlang mountainous chickens and AA broilers were randomly divided into uninfected group and infected group. Every group has fifty chickens. Each chicken in the infected group was infected intraperitoneally with 1500 PFU of virulent MDV J-1 strain. The control group was mock infected with viral diluents. The MDV-infected group was kept under identical condition as the uninfected age-matched control. 

At 4, 7, and 21 d.p.i, six broilers and eight Erlang mountainous chickens of each group were euthanized, and lymphoid tissues including spleen, thymus, and bursa of Fabricius were collected from euthanized chickens. Collected samples were snap frozen in liquid nitrogen and then stored at −80°C. Meanwhile, the rest of chickens in infected group were monitored for death until 21 d.p.i.

### 2.3. DNA and RNA Extraction and cDNA Synthesis

Total RNA was isolated from spleen, thymus, and bursa of Fabricius of infected and uninfected chicken by using TRIZOL reagent (Invitrogen Co., Ltd, Beijing, China) according to the manufacturers' instructions. Extracted RNA was dissolved into 40 *μ*L RNase-free water and stored at −80°C until used. 

DNA was extracted from spleen of MDV-infected chickens by TRIZOL reagent (Invetrogen Co., Ltd, Beijing, China) according to manufacturer's protocol and was dissolved in TE buffer, as well as stored at −20°C until used.

Reverse transcription of total RNA was carried out using PrimeScript RT reagent Kit (TAKARA, Dalian, China) according to the manufacturers' instructions. The reaction was performed in a volume of 20 *μ*L containing 4 *μ*L of 5 × PrimeScript Buffer, 1 *μ*L of PrimeScript RT Enzyme Mix I, 1 *μ*L of Oligo dT Primer, 1 *μ*L of Random 6 mers, 11 *μ*L of RNase-free water, and 2 *μ*L of total RNA. The reaction was done at 37°C for 15 min and 85°C for 5 sec. The synthesized cDNAs preparation was stored at −20°C until used in the real-time PCR.

### 2.4. Primer Design

The absolute MDV genome load in the MDV-infected chicken's spleen was quantified using primers specific for MDV-*meq* gene. The primers specific for *meq MDA-5, IRF-3, IFN-*α** and *IFN-*β**, as well as *GAPDH* genes were designed by Primer 5.0 and used for relative quantification of gene expression in collected tissues. The specificity of the primers was confirmed by using BLAST program in NCBI. The sequence and parameters of primers were shown in Table 1.

### 2.5. Construct for Standard Curve

The real-time PCR for relative quantification of the target genes expression was performed using the standard curve. The fragment of target gene was PCR amplified using the specific primers. The condition of amplification included an initial heat denaturing at 94°C for 4 min, 30 cycles of 94°C for 30 s, 55°C for 30 s, 72°C for 2 min. PCR products were tested in the 1.5% agarose gel and cloned into the p-vector (TAKARA, Dalian, China). The plasmid DNA of target and reference genes was 10-fold serial diluted (10^−1^ to 10^−9^) and was used to generate standard curves on the CFX96 real-time PCR according to the following PCR condition.

### 2.6. Real-Time PCR

The expression levels of target gene were detected by using the SsoFast-Evagreen assay on the CFX96 real-time PCR thermal cycle instrument (Bio-Rad). Dilution of the standards was used as calibrator in each real-time PCR assay. PCR reaction mixture of 20 *μ*L contained 10 *μ*L of SsoFast Evagreen (Bio-Rad), 1 *μ*L of each specific primer, 6 *μ*L of ddH_2_0, and 2 *μ*L of cDNA. All Real-time PCR reaction was carried out in the triplicate for each sample. The thermal cycling conditions consisted of an initial heat denaturing at 98°C for 2 min, 39 cycles of 98°C for 2 s, and optimal annealing temperature of each primer pair for 15 s. Melting-curve analyses were applied in each amplification to test the specificity of amplification.

### 2.7. Statistical Analysis

The efficiency of real-time PCR (*E*) was calculated by 10^(−1/slope  of  the  standard  curve)^, and the level of mRNA expression of target gene was calculated relative to *GAPDH* gene expression and was expressed as ratios. The formula used to quantify the relative amount of gene expression was 2^−ΔCT^. The absolute numbers of MDV genome per 100 ng of spleen DNA were calculated based on standard curve. The MDV genome load data and target gene expression data were subjected to *t*-test. *T*-test and comparisons were considered significant at *P* < 0.05.

## 3. Results

### 3.1. Generation of Standard Curves

Standard curves for relative quantification of *MDA-5, IRF-3, IFN-*α** and *IFN-*β**, and *GAPDH* gene were generated, and *GAPDH* was used as reference gene. The amplification efficiency of *MDA-5, IRF-3, IFN-*α**, *IFN-β*, and *GAPDH* was 101.9%, 96%, 96.7%, 100.2%, and 99.4%, respectively.

### 3.2. The Mortality of Two-Breed Chickens after Being Infected with MDV

**Figure 1:**
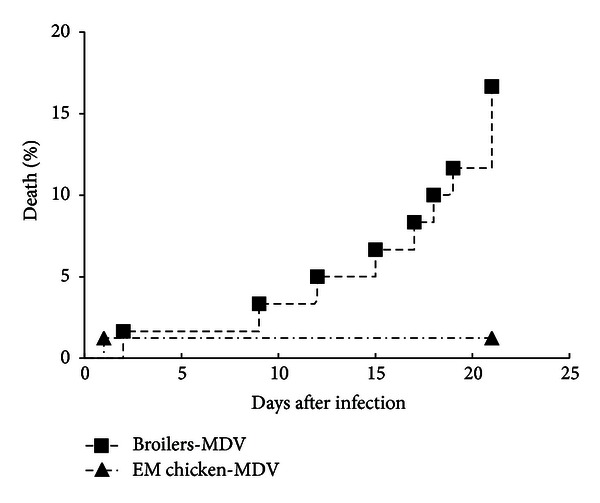
The death rate of two-breed chickens following infection with MDV. The groups were as follows: Broiler-MDV= chickens from broilers group infected with MDV, EM chicken-MDV = chickens from Erlang mountainous chickens group infected with MDV.

After being infected with MDV, the death of two-breed chickens was monitored and the data are shown in Figure 1. We found that the mortality of broilers was higher than Erlang mountainous chickens at the same condition upon infection with MDV, and the death rate of broilers had a gradually increasing trend from 9 d.p.i to 21 d.p.i. But the death of Erlang mountainous chickens had not presented in the phase. These results indicate that the Erlang mountainous chicken have more resistance to MDV than broiler.

### 3.3. MDV Genome Load in the Spleen of MDV-Infected Broilers and ErLang Mountainous Chickens

**Figure 2:**
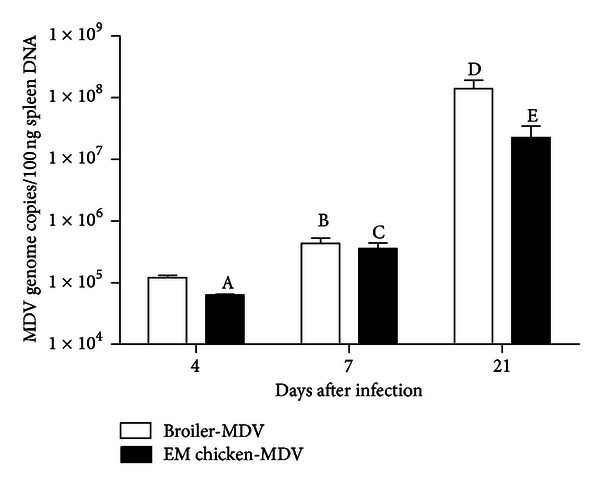
MDV genome load in spleen of broilers and Erlang mountainous chickens following infection with MDV. The groups were as follows: Broiler-MDV = chickens from the broilers infected with MDV, EM chicken-MDV=chickens from Erlang mountainous chickens infected with MDV. MDV infected group was infected with virulent strain of MDV. At 4, 7, and 21 d.p.i, six broilers and eight Erlang mountainous chickens of infected group were killed. The MDV genome loads in spleen of killed chickens were analyzed by real-time PCR. A: significant compared to broiler-MDV observed at 4 d.p.i. B: and C: significant compared to broiler-MDV and EM chicken-MDV observed at 4 d.p.i, respectively. D: significant compared to broiler-MDV observed at 7 d.p.i. E: significant compared to broiler-MDV observed at 21 d.p.i. Error bars represent standard error of the mean.

Spleen DNA extracted from MDV-infected chickens was analyzed by real-time PCR and the result is shown in Figure 2. MDV genome could be detected in all infected chickens, whereas uninfected-control chickens did not show any amplification of *Meq* gene. After infection with MDV, the MDV genome load in the spleens of broilers and Erlang mountainous chickens had a gradually increasing trend from 4 d.p.i to 21 d.p.i. The MDV genome load in spleens of broilers and Erlang mountainous chickens was significantly higher at 7 d.p.i when compared to that in spleens of the same line at 4 d.p.i (*P* = 0.0076 and *P* = 0.0082), respectively. In broilers, it was also significantly higher at 21 d.p.i than that at 7 d.p.i (*P* = 0.0494). Meanwhile, the MDV genome load in spleens of broilers was significantly higher than that in Erlang mountainous chickens at 4 d.p.i (*P* = 0.003) and 21 d.p.i (*P* = 0.038). These results suggest that Erlang mountainous chicken might have more capability of controlling MDV replication *in vivo*. 

### 3.4. Detection of MDA-5, IRF-3, IFN-*α*, and IFN-*β* Genes in Spleens of MDV-Infected and MDV-Uninfected Chickens

**Figure 3:**
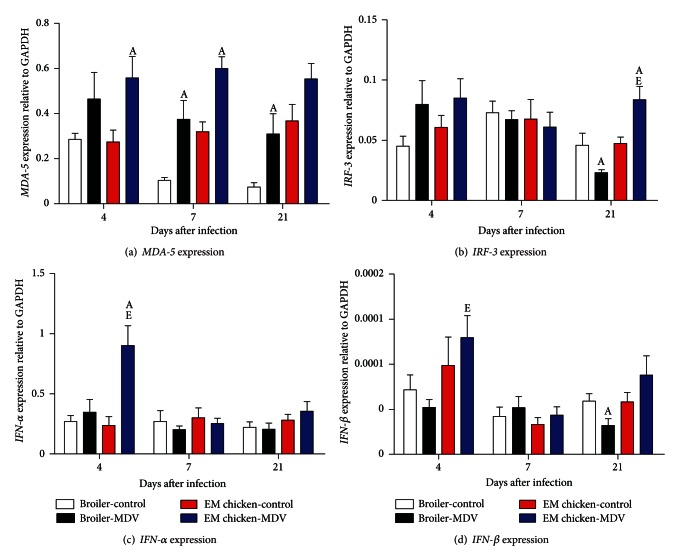
Expression of *MDA-5* (a), *IRF-3* (b), *IFN-*α** (c), and *IFN-*β** (d) genes in spleen of chicken infected with virulent of MDV or uninfected control chickens. The groups were as follows: Broiler-control = uninfected broilers, Broiler-MDV = MDV-infected chickens of broilers, EM chicken-control = uninfected chickens of Erlang mountainous chicken, and EM chicken-MDV = MDV-infected chickens of Erlang mountainous chicken. At 4, 7, 21 d.p.i, six broilers and eight Erlang mountainous chickens of each group were killed. The expression of genes in spleen of every killed chicken was analyzed. Error bars represent standard error of the mean. A: significant difference comparing MDV-infected chickens with uninfected chickens of the same line at the same point. E: significant difference comparing MDV-infected Erlang mountainous chicken with MDV-infected broilers at same point.

The expression of *MDA-5* gene in spleens is shown in Figure 3(a). The expression of *MDA-5* gene had an increasing trend in spleens of both two-breed chickens infected with MDV compared to uninfected chickens. At 7 and 21 d.p.i, the MDV-infected broilers have significantly higher *MDA-5* mRNA expression in spleens compared to the uninfected-control same line (*P* = 0.0117 and *P* = 0.0343). Meanwhile, the expression of this gene in Erlang mountainous chickens had a dramatic rise compared to the uninfected-control same line at 4 and 7 d.p.i (*P* = 0.0207 and *P* = 0.0027).

The expression of *IRF-3* gene was observed in spleens of broilers and Erlang mountainous chickens (Figure 3(b)). It had a slightly increasing trend in spleens of two-breed chickens at 4 d.p.i, while the trend was not significant. However, at 21 d.p.i, the expression of this gene in the spleens of MDV-infected broilers was significantly lower than the uninfected ones (*P* = 0.0375). By contrast, the expression of the gene was significantly higher in the spleens of MDV-infected Erlang mountainous chickens than the uninfected ones (*P* = 0.0212). And the expression of* MDA-5* gene in spleens of MDV-infected broilers at 21 d.p.i was significantly lower when compared to that in spleens of MDV-infected Erlang mountainous chickens (*P* = 0.0006).

The expression of *IFN-*α** and *IFN-*β** in spleen was shown in Figures 3(c) and 3(d), respectively. The expression of *IFN-*α** in spleen of MDV-infected Erlang mountainous chickens was significantly higher when compared to that in spleen of the uninfected-control same line and MDV-infected broilers at 4 d.p.i (*P* = 0.0075 and *P* = 0.0179). Even though the expression of *IFN-*β** gene increased moderately in spleen of Erlang mountainous chickens during infection with MDV, the difference was not significant, and expression of this gene in spleen Erlang mountainous chickens was significantly higher than that in spleen of MDV-infected broilers at 4 d.p.i (*P* = 0.011). Interestingly, the expression of *IFN-*β** gene had a substantial decrease in spleen of MDV-infected boiler chickens at 21 d.p.i (*P* = 0.0428).

### 3.5. The Expression of MDA-5, IRF-3, IFN-*α* and IFN-*β* in Thymus of MDV-Infected and MDV-Uninfected Chickens

**Figure 4:**
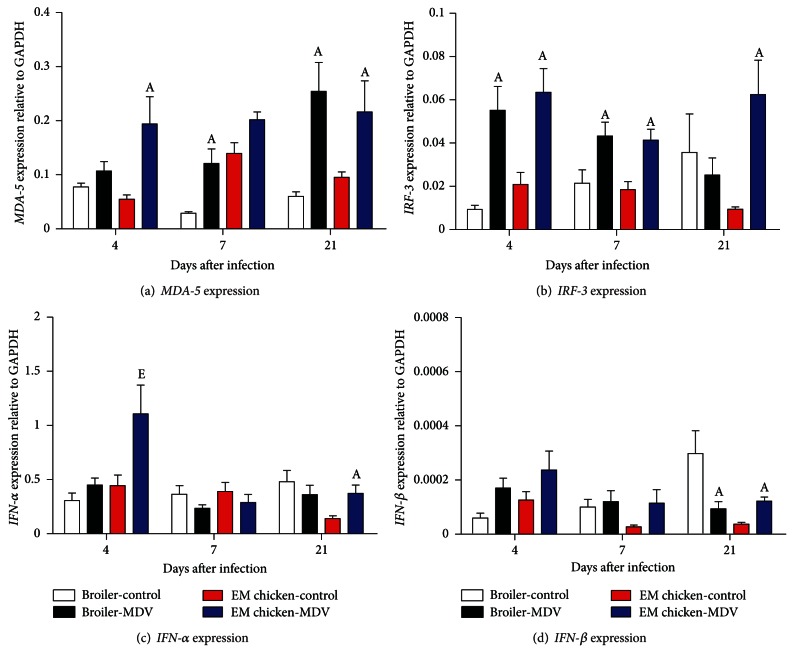
Expression of *MDA-5* (a), *IRF-3* (b), *IFN-*α** (c), and *IFN-*β** (d) genes in thymus of chicken infected with virulent of MDV or uninfected control chickens. The groups were as follows: Broiler-control = uninfected broilers, Broiler-MDV = MDV-infected chickens of broilers, EM chicken-control = uninfected chickens of Erlang mountainous chicken, and EM chicken-MDV = MDV-infected chickens of Erlang mountainous chicken. At 4, 7, and 21 d.p.i, six broilers and eight Erlang mountainous chickens of each group were killed. The expression of genes in thymus of every killed chicken was analyzed. Error bars represent standard error of the mean. A: significant difference comparing MDV-infected chickens with uninfected chickens of the same line at the same point. E: significant difference comparing MDV-infected Erlang mountainous chicken with MDV-infected broilers at the same point.

The expression of *MDA-5* in thymus was shown in Figure 4(a). MDV infection caused upregulation of expression of *MDA-5* gene in thymus of broilers and Erlang mountainous chickens. At 7 and 21 d.p.i, MDV-infected broilers had significantly higher expression of *MDA-5* gene in thymus than the uninfected-control same line (*P* = 0.0068 and *P* = 0.0102). Furthermore, the expression of *MDA-5* gene in the thymus of MDV-infected Erlang mountainous chickens was also significantly higher than the uninfected-control same line at 4 and 21 d.p.i (*P* = 0.0344 and *P* = 0.0242).

The expression of *IRF-3* in thymus was shown in Figure 4(b). After infection with MDV, the expression of *IRF-3* gene was significantly higher in the thymus of the MDV-infected broilers when compared to that in the thymus of the control-uninfected broilers at 4 d.p.i (*P* = 0.0112) and 7 d.p.i (*P* = 0.0344). However, the expression of *IRF-3* gene was significantly higher in the thymus of Erlang mountainous chickens when compared to uninfected-control same line at 4 d.p.i (*P* = 0.0138), 7 d.p.i (*P* = 0.0029), and 21 d.p.i (*P* = 0.0021), respectively.

The expression data for *IFN-*α** and *IFN-*β** in spleen were shown in Figures 4(c) and 4(d), respectively. The expression of *IFN-*α** in thymus of Erlang mountainous chickens has an increased tendency at 4 and 21 d.p.i, and the increased tendency reached significantly only at 21 d.p.i (*P* = 0.0085). Meanwhile the expression of *IFN-*α**in thymus of MDV-infected Erlang mountainous chickens was significant higher when compared to that in the thymus of MDV-infected broilers (*P* = 0.0314). In addition, MDV infection caused the increase of expression of *IFN-*β** in the Erlang mountainous chickens, and the increased trend reached significant at 21 d.p.i (*P* = 0.0001). By contrast, the expression of *IFN-*β** in the thymus of MDV-infected broilers decreased significantly when compared to uninfected broilers at 21 d.p.i (*P* = 0.0251).

### 3.6. The Expression of MDA-5, IRF-3, IFN-*α* and IFN-*β* Genes in Bursa of Fabricius of MDV-Infected and Uninfected Chickens

**Figure 5:**
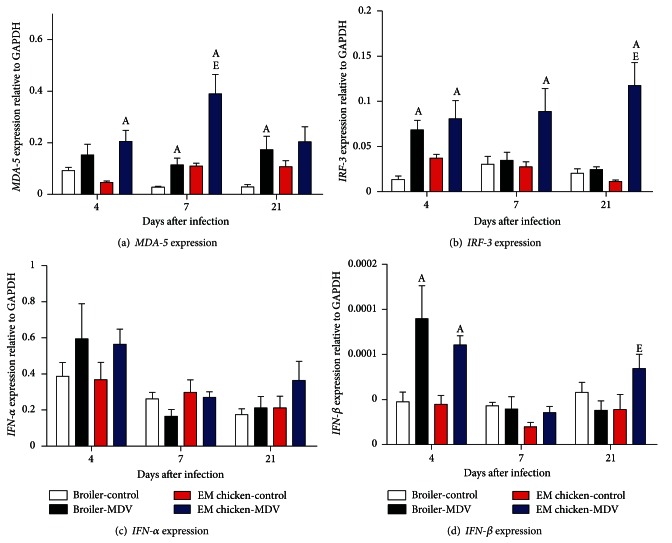
Expression of *MDA-5* (a), *IRF-3* (b), *IFN-*α** (c), and *IFN-*β** (d) genes in bursa of Fabricius of chicken infected with virulent of MDV or uninfected control chickens. The groups were as follows: Broiler-control = uninfected broilers, Broiler-MDV = MDV-infected chickens of broilers, EM chicken-control = uninfected chickens of Erlang mountainous chicken, and EM chicken-MDV = MDV-infected chickens of Erlang mountainous chicken. At 4, 7, and 21 d.p.i, six broilers and eight Erlang mountainous chickens of each group were killed. The expression of genes in bursa of Fabricius of every killed chicken was analyzed. Error bars represent standard error of the mean. A: significant difference comparing MDV-infected chickens with uninfected chickens of the same line at the same point. E: significant difference comparing MDV-infected Erlang mountainous chicken with MDV-infected broilers at the same point.

The expression of *MDA-5* in bursa of Fabricius was shown in Figure 5(a). The expression of *MDA-5* gene in bursa of Fabricius of both two breeds had a rising trend following infection with MDV, which approached significant in broilers at 7 and 21 d.p.i (*P* = 0.0077 and *P* = 0.0185) and in Erlang mountainous chickens at 4 and 7 d.p.i (*P* = 0.0042 and *P* = 0.0059).

The expression of *IRF-3* in bursa of Fabricius was shown in Figure 5(b). The Erlang mountainous chickens infecting with MDV showed significant increase in expression of *IRF-3* in bursa of Fabricius tissues when compared to that in control-uninfected chickens at 4 d.p.i (*P* = 0.0438), 7 d.p.i (*P* = 0.0345), and 21 d.p.i (*P* = 0.0009). However, the significant increase in the expression of this gene of MDV-infected broilers occurred only at 4 d.p.i (*P* = 0.0011).

The expression data for *IFN-*α** and *IFN-*β** in bursa of Fabricius were shown in Figures 5(c) and 5(d), respectively. The expression of *IFN-*β** gene in bursa of Fabricius of two breeds both was significantly higher than that in the uninfected same line at 4 d.p.i (*P* = 0.0231 and *P* = 0.0013), respectively. Although the expression of *IFN-*β** revealed a sharp rise in the bursa of Fabricius of Erlang mountainous chickens infecting with MDV, it did not approach significant (*P* = 0.0892). Moreover, it was obtained that the expression of this gene was significantly higher in bursa of Fabricius of Erlang mountainous chickens than that in broilers at 21 d.p.i. (*P* = 0.0398).

## 4. Discussion

It has been proved that the resistance of chicken to MDV is influenced by different genetic backgrounds [29]. And the chicken's different haplotypes of major histocompatibility complex (MHC) affect the resistance of chicken to disease. It have been demonstrated that the B21 and B19 haplotypes are associated with resistance and susceptibility MDV, respectively [30]. Meanwhile, several quantitative trait loci (QTL) against to MDV within the chicken's genome had been identified using genetic markers [31–33]. However, the underlying mechanism how genetic background influences the resistance of chicken to MDV remains unknown. In this study, two breeds, economic line-broilers and native line-Erlang mountainous chickens, were adopted for being infected with MDV. Broilers used in our experiment is special breed for meat production through a long-time high-intensity selection, and it has a higher growth speed in muscle tissue. On the contrary, Erlang mountainous chicken is a native breed, which have not been selected for a long time for any economic trait. After infection with MDV, Erlang mountainous chickens showed more resistance to MDV infection than broilers. It is indicated that overselection for economic trait indeed influence the resistance of chicken response to MDV infection. Previous study showed that the second cytolytic infection induced by MDV occurred in the susceptible chickens from approximately 18 d.p.i onward [29]. In our experiment, the death of broiler mainly occurred from 16 d.p.i to 21 d.p.i, and we speculated that the death of broilers might be the consequent of MDV-mediated second cytolytic infection during this phase.

 Although both genetically susceptible and resistant chickens can be infected with MDV, genetically resistant chickens are capable of controlling the MDV genome load in spleens and feather [26, 34]. In agreement with this, in the current study, the MDV genome load appeared in spleens of MDV-infected two-breed chickens, and the MDV genome load in spleen of broilers was significantly higher when compared to Erlang mountainous chickens at 4 and 21 d.p.i. These results further indicate that genetic background function as crucial element for affecting MDV genome load in chicken.

It has been proved that RLR-mediated immune pathway mainly is involved in detection and response to RNA virus [35]. However, little is known about the exact role of RLR-mediated innate immune *in vivo* response to DNA virus. Due to the deficiency of RIG-I in chicken, chicken serve as a good animal modern for studying the role of MDA-5 *in vivo* response to DNA virus. 

In our study, the expression of *MDA-5* gene was induced in three immune tissues of two-breed chickens at 4, 7, and 21 d.p.i. It is suggested that MDA-5 might be involved in detection and response against MDV. Because MDV belongs to DNA virus, how does chicken utilize MDA-5 to detect MDV? The study in human primary macrophages found that MDA-5 is responsible for recognition of HSV-1, and the process is dependent on viral replication [36]. Owing to dsRNA generated by positive-strand RNA viruses and DNA viruses during viral replication [37], we deduce that dsRNA produced by MDV during replication might serve as resources which are detected byMDA-5 and trigger RLR-mediated immune pathway. Meanwhile, some studies revealed that RNA polymerase III was involved in detection of cytosolic DNA and triggering production of type I in human cell, and inhibition of RNA polymerase III also blocked production of interferon induced by DNA virus, such as Herpes simplex virus-1 (HSV-1) and Epstein-Barr virus (EBV) [38–40]. However, the involvement of polymerase III in DNA virus is dependent on RIG-I-mediated immune pathway, independent on MDA-5. Owing to the absence of *RIG-I* in chicken, further study is needed to investigate whether chicken polymerase III and MDA-5 coordinately detect MDV and promote the expression of interferon at cell level.

Chicken IRF-3 was firstly identified as the first example of a nonmammalian interferon regulatory factor [41], but it was thought as the homology of human IRF-7 due to its higher DNA sequence homology with human IRF-7, rather than human *IRF-3* [42]. Mammalian IRF-3 is mainly responsible for induction of *IFN-*β** gene but not the *IFN-*α**, yet *IRF-7* efficiently activated both *IFN-*α** and *IFN-*β** [43, 44]. In our experiment, we found that expression level of *IRF-3* was associated with the expression of *IFN-*α** and *IFN-*β**. It is suggested that chicken IRF-3, like human IRF-7, is also responsible for the expression *IFN-*α** and *IFN-*β** in chicken. 

Previous study indicated that vaccinating with MDV vaccine could enhance the expression of the *IRF-3* gene in chicken during latent period of MDV infection [45]. And the role of interferon chicken response to MDV infection had been proved [24, 46]. In the present study, we discovered that the expression of both *IRF-3* and* IFN-*β** genes had been downregulated in spleen and thymus of broiler at 21 d.p.i, but it showed an upregulation in Erlang mountainous chickens. Owing to the death of broilers observed in this phase, these results further highlight the role of interferon in chicken response against MDV infection. Meanwhile, these results further support the previous conclusion that expression pattern of interferon and cytokine was correlated with genetic background of chicken during MDV infection [26, 28, 34]. Besides, giving that the MDV-mediated secondly cytolytic replication might be occurred in chicken during this phase, we speculate that the change of these genes expression in broiler is the result of MDV-mediated secondly cytolytic replication which causes immunosuppression in broilers for inhibition of interferon expression. These results further suggest that the downregulation of expression of *IRF-3* and interferon gene also might be associated with MDV reactivation. If we could explore deeply the mechanism that MDV infection causes immunosuppression in susceptible chicken, it will make us better understand the interaction between viruses and host.

## 5. Conclusions

In summary, our study found that the expression of *MDA-5* gene was induced in chicken following infection with MDV, which suggested that MDA-5 might be involved in recognition of MDV in chicken. Importantly, we observed the different expression pattern of *IRF-3* and *IFN-*β** genes in broilers and Erlang mountainous chickens at 21 d.p.i. We conclude that it might be affected by genetic background which serve as the main reason leading to the different resistance of two-breed response against MDV infection. Further study is required to elucidate the underlying mechanism between host innate immune and different genetic backgrounds.

## Figures and Tables

**Table 1:** Genes and primer pairs used in this study.

Genes		Primer pairs sequences (5′–3′)	Annealing temperatures (°C)	Amplicons (bp)	Accession numbers
Meq	Forward Reverse	ACGCAGGGAGCAGACGTACTAT CCATAGGGCAAACTGGCTCAT	63°C	155	YP_001033993
MDA-5	Forward Reverse	GTTGCTGTAGGAGATGCAAGTG ATCTGGCTCAGGTGAAGCTCT	60°C	114	NM_001193638
IRF-3	Forward Reverse	TACACTGAGGACTTGCTGGAGGT AAGATGGTGGTCTCCTGATCC	62°C	170	NM_205372
IFN-*α*	Forward Reverse	CAGGATGCCACCTTCTCTCAC AGGATGGTGTCGTTGAAGGAG	60°C	113	NM_205427
IFN-*β*	Forward Reverse	CCTCAACCAGATCCAGCATTAC CCCAGGTACAAGCACTGTAGTT	59°C	167	NM_001024836
GAPDH	Forward Reverse	AGGACCAGGTTGTCTCCTGT CCATCAAGTCCACAACACGG	62°C	153	NM_204305
